# Telepsychiatry adoption across hospitals in the United States: a cross-sectional study

**DOI:** 10.1186/s12888-021-03180-8

**Published:** 2021-04-07

**Authors:** Zhong Li, Sayward E. Harrison, Xiaoming Li, Peiyin Hung

**Affiliations:** 1 Department of Health Services Policy and Management, Arnold School of Public Health, University of South Carolina, 915 Greene St., Suite, Columbia, SC 348 USA; 2 Department of Health Management, School of Medicine and Health Management, Tongji Medical College, Huazhong University of Science and Technology, Wuhan, Hubei China; 3 South Carolina SmartState Center for Healthcare Quality, Arnold School of Public Health, University of South Carolina, Columbia, SC USA; 4 Department of Psychology, College of Arts and Sciences, University of South Carolina, Columbia, SC USA; 5 Department of Health Promotion, Education, and Behavior, Arnold School of Public Health, University of South Carolina, Columbia, SC USA; 6 Rural and Minority Health Research Center, University of South Carolina, Columbia, SC USA

**Keywords:** Telepsychiatry, Telemedicine, Hospital psychiatry, Access to care, Continuity of care

## Abstract

**Background:**

Access to psychiatric care is critical for patients discharged from hospital psychiatric units to ensure continuity of care. When face-to-face follow-up is unavailable or undesirable, telepsychiatry becomes a promising alternative. This study aimed to investigate hospital- and county-level characteristics associated with telepsychiatry adoption.

**Methods:**

Cross-sectional national data of 3475 acute care hospitals were derived from the 2017 American Hospital Association Annual Survey. Generalized linear regression models were used to identify characteristics associated with telepsychiatry adoption.

**Results:**

About one-sixth (548 [15.8%]) of hospitals reported having telepsychiatry with a wide variation across states. Rural noncore hospitals were less likely to adopt telepsychiatry (8.3%) than hospitals in rural micropolitan (13.6%) and urban counties (19.4%). Hospitals with both outpatient and inpatient psychiatric care services (marginal difference [95% CI]: 16.0% [12.1% to 19.9%]) and hospitals only with outpatient psychiatric services (6.5% [3.7% to 9.4%]) were more likely to have telepsychiatry than hospitals with neither psychiatric services. Federal hospitals (48.9% [32.5 to 65.3%]), system-affiliated hospitals (3.9% [1.2% to 6.6%]), hospitals with larger bed size (Quartile IV vs. I: 6.2% [0.7% to 11.6%]), and hospitals with greater ratio of Medicaid inpatient days to total inpatient days (Quartile IV vs. I: 4.9% [0.3% to 9.4%]) were more likely to have telepsychiatry than their counterparts. Private non-profit hospitals (− 6.9% [− 11.7% to − 2.0%]) and hospitals in counties designated as whole mental health professional shortage areas (− 6.6% [− 12.7% to − 0.5%]) were less likely to have telepsychiatry.

**Conclusions:**

Prior to the Covid-19 pandemic, telepsychiatry adoption in US hospitals was low with substantial variations by urban and rural status and by state in 2017. This raises concerns about access to psychiatric services and continuity of care for patients discharged from hospitals.

**Supplementary Information:**

The online version contains supplementary material available at 10.1186/s12888-021-03180-8.

## Background

Over 46 million Americans experienced a mental illness [1]; however, less than half (42.9%) of these individuals received mental health services in a 12-month window, partially due to stigma and limited access to care [1]. Every year, millions of emergency department (ED) visits in the United States (US) involve mental illness and substance use disorders [2]. Recent data from California indicate that nearly 30% patients seen in a ED had a prior mental health diagnosis [3]. The number of ED visits for primarily mental health reasons has increased markedly over past two decades [4]. For US adults under age 45, mental illness is the top reason for hospitalization [5]. In 2016, 7.7 million hospitalizations, accounting for 21.7% of national hospital stays, were attributable to mental and substance use disorders [6]. Linkage to and continuity of mental health care is a challenge, with persistent high suicide rates for mental health patients following hospital discharge [7].

Access to psychiatric care in the US is inequitable across communities due in part to an uneven distribution of psychiatrists, psychologists, and other mental health professionals [8]. A shortage of psychiatrists is more likely to occur in non-metropolitan counties than metropolitan counties; in 2015, nearly 70% of counties in the West North Central Census Division lacked a licensed psychiatrist—a rate over tenfold higher than the New England Census Division where only 6% of counties had no psychiatrist [8]. Amid provider shortages, the national number of psychiatric beds declined from 0.31 to 0.21 beds per 1000 population from 2000 to 2016 [9]. Limited access to psychiatric inpatient care may result in EDs being overcrowded with patients with psychiatric conditions [10], creating challenges for patient safety and increasing health care costs [11, 12]. Meanwhile, lack of or loss to follow-up after psychiatric inpatient discharge can also lead to non-adherence to medications [13], readmission among high-risk patients, and even suicidality [14]. Specifically, the period between psychiatric discharge and follow-up is a particularly critical time for suicidal risk, with high rates of suicides occurring within one week of hospital discharge for psychiatric patients [15]. However, in 2015, only 55.8% of hospitals delivered timely care following psychiatric hospitalizations; psychiatric specialty hospitals (52.9%) and publicly owned hospitals (52.3%) had lower follow-up rates within 30 days post discharge than their non-specialty (57.0%) and private hospital counterparts (59.2%), respectively [16].

Mental health advocates have promoted telepsychiatry as one possible solution to the shortage of mental health professionals [17–19]. Within a hospital setting, telepsychiatry may enable providers to complete virtual psychiatric evaluations, provide teletherapy, communicate briefly and check-in with patients, and offer patient education [20, 21]. In addition, telepsychiatry may overcome some barriers to care by enabling patients to access mental health services from a private, trusted location. Telepsychiatry may also be an effective way to decrease ED visits for non-life-threatening mental health conditions and to ensure continuity of care for patients after being discharged from inpatient care. These virtual care systems have been proven to be well received by patients, associated with decreased admissions or readmissions to psychiatric hospitals, and cost effective [22]. In the wake of a pandemic like the coronavirus disease 2019 (COVID-19), telepsychiatry becomes a critical tool to cope with the growing needs for mental health care, while allowing for social distancing practices [23, 24].

While telehealth adoption has steadily increased nationwide, telepsychiatry has lagged behind [17, 18]. From 2010 to 2017, the proportion of hospitals with any telehealth capacity doubled from 35 to 76% in the US [25]. Despite well-documented acceptance of telepsychiatry by patients and clinicians and higher perceived efficacy than standard care by patient populations [26–28], only about 20% of hospital-based EDs had telepsychiatry as of 2016 [29]. In 2013, less than 1% of rural Medicare beneficiaries reported ever having utilized telehealth for mental health services [30]. Telepsychiatry use was also uncommon among commercially insured populations, with only 0.5 telepsychiatry visits per 1000 members per quarter in 2017 [31]. Even among mental health facilities, only about 30% offered telepsychiatry by 2017 [32]. Full implementation of telepsychiatry takes months, even for well-resourced hospitals [21]. As community mental health services have been reduced over the past decades [33], hospital-based services play an increased role in preventing and managing psychiatric crises, especially for patients with severe mental health disorders or being discharged from inpatient care. Assessing the distribution of telepsychiatry adoption across hospital settings is an essential first step to understand the availability of services and to identify areas and settings where gaps exist.

Prior work has demonstrated geographic variations in the availability of mental health resources across counties nationwide [34–36], and variations in telepsychiatry adoption in mental health facilities [32]. However, to the best of our knowledge, no study has examined geographic distribution of telepsychiatry adoption across US hospitals. Much remains unknown about characteristics of hospitals that choose to adopt telepsychiatry, which is vital to identify where the shortages of telepsychiatry are and where to enact policies to support more rapid telepsychiatry adoption. Therefore, this study aimed to assess the geographic distribution of telepsychiatry across hospitals in the US and investigate characteristics associated with telepsychiatry adoption.

## Methods

### Data sources

This study used three datasets to derive hospital- and county-level characteristics documented by previous literature [37]: 1) the 2017 American Hospital Association (AHA) Annual Survey, 2) the Area Health Resource File, and 3) hospital-level financial performance data from the Centers for Medicare & Medicaid Services Healthcare Cost Report Information System. In 2017, the AHA Annual Survey had a hospital response rate of 85%. In this study, we initially included 4602 acute care hospitals located in 50 states and Washington, DC that provided general medical or surgical care, psychiatric services, or pediatric psychiatric care. Hospitals in US territories were excluded due to large variations in policies and regulations. We then further excluded 1127 hospitals that did not provide a response to whether they had telepsychiatry, yielding 3475 acute care hospitals in the final data set. Compared to the 1127 excluded hospitals, included hospitals were more likely to have no inpatient or outpatient psychiatric services, to be federal hospitals or private non-profit hospitals, to be teaching hospitals, to be less profitable, and to be in counties with high uninsured rates and high poverty rates (Additional file 1: Table 1).

### Measures

The primary outcome is whether a hospital adopted telepsychiatry in 2017. According to the AHA survey, telepsychiatry is a type of telehealth defined as “a broader variety technologies and tactics to deliver virtual diagnosis and management, education, and other health care with telecommunications technologies” [38]. In particular, telepsychiatry is considered to “involve a range of services including psychiatric evaluation, therapy, patient education, and medication management” [38].

#### Hospital characteristics

Hospital variables included ownership (federal, non-federal public, private for-profit, and private not-for-profit), teaching status, system affiliation, designation as critical access hospital, hospital beds staffed, ratio of Medicaid inpatient days to total inpatient days, provision of in-person psychiatric services, and profit margins. In the US, federal hospitals, funded by the federal government, typically handle the healthcare and medical needs of select populations such as veterans. Non-federal public hospitals are generally funded by state and city governments. For-profit hospitals earn profits that go to shareholders; while private not-for-profit hospitals often receive tax exemptions that are unavailable to for-profit hospitals [39]. Private hospitals often have access to latest technologies and equipment, and hospital owners and administrators determine the budget, financing and regulation compliance [40]. Hospitals may be freestanding or affiliated with a health system (i.e., system affiliated hospitals).

#### County characteristics

Counties were grouped into urban, rural micropolitan, and rural noncore areas, based on the Urban Influence Codes created by the Office of Management and Budget. Metropolitan (urban) areas include central counties with one or more urbanized areas – densely-settled urban entities with 50,000 or more people – and outlying counties with at least 2% of labor force commuting to a central metropolitan county. Nonmetro counties outside the boundaries of metro areas are categorized by population density into micropolitan (counties with an urbanized area of 10,000–49,999 residents) and noncore counties (all other counties) [41].

To identify county-level factors associated with telepsychiatry adoption, we included annual median household income, age groups, racial distribution of residents, rates of uninsured residents, unemployment rates, rates of population living in poverty (defined as ≤200% federal poverty line), whether the county was designated as a mental health professional shortage area, and total number of psychiatrists in the county.

### Statistical analyses

We first mapped hospitals with telepsychiatry based on the latitudes and longitudes of their address using SAS version 9.4 [42]. Chi-squared tests and Kruskal-Wallis rank-sum tests were used to compare hospital- and county-level characteristics across hospitals with and without telepsychiatry. Generalized logistic regression models were used to estimate marginal associations of each predictor on telepsychiatry adoption, with county-level clustering. Multicollinearity was assessed using variance inflation factors (VIF) that did not indicate the presence of multicollinearity among predictors (i.e., VIF = 2.90). We selected the final model based on the lowest values of Akaike Information Criterion and Bayesian Information Criterion [43]. The final model included hospital location, provision of psychiatric services, ownership, system affiliation, hospital beds staffed, ratio of Medicaid inpatient days to total inpatient days, profit margins, county-level age group, race/ethnicity, rate of population uninsured, designation as mental health professional shortage areas, number of psychiatrists, and census region (Additional file 1: Tables 2–4). We also conducted sensitivity analyses by replacing county-level uninsured rate with county-level unemployed rate and rate of population living in poverty. All analyses were conducted using SAS version 9.4 and Stata version 14.0.

## Results

### Distribution of telepsychiatry adoption across US hospitals

**Fig. 1:**
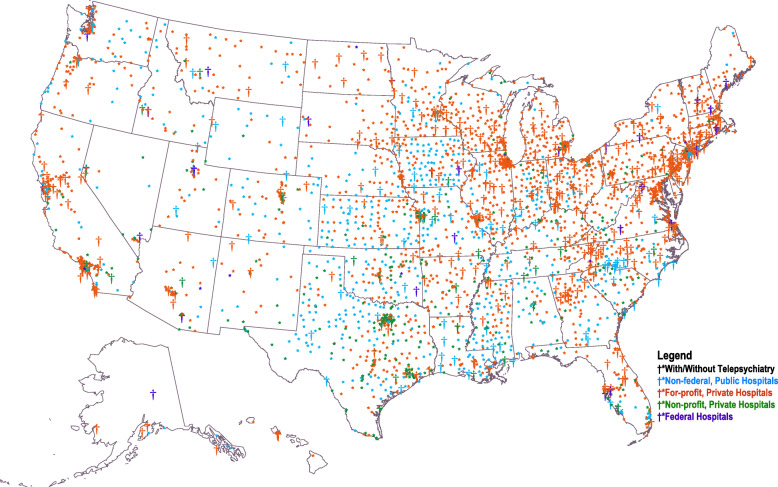
Telepsychiatry adoption by hospital ownership in 2017. Sources: Data on telepsychiatry were derived from 2017 AHA Annual Survey dataset. Telepsychiatry can deliver a range of services including psychiatric evaluation, therapy, patient education, and medication management. The map we used to demonstrate telepsychiatry adoption by hospital ownership in 2017 was provided by the licensed SAS/GRAPH; Most of the map data sets provided with SAS/GRAPH contain geographic area (boundaries) represented in terms of longitude and latitude, x and y coordinates respectively

Figure 1 illustrates the geographic distribution of telepsychiatry adoption across hospitals in the US. Of 3475 hospitals, approximately 16% reported having telepsychiatry in 2017 (Table 1). Only 19.4% of urban hospitals had adopted telepsychiatry, and far fewer hospitals in rural micropolitan (13.6%) and rural noncore areas (8.3%) did. However, approximately 32.3% (267 of 827) of urban counties had at least one hospital with telepsychiatry, compared to 16.0% (78 of 489) of rural micropolitan and 9.4% (69 of 737) of rural noncore counties. Hospitals in affluent counties (lower proportions of residents living in poverty or uninsured residents) were more likely to adopt telepsychiatry compared with hospitals in less affluent counties. Hospitals in counties designated as mental health professional shortage areas, with smaller number of psychiatrists, also reported lower levels of telepsychiatry adoption than their counterparts. The proportion of telepsychiatry adoption varied significantly by state (Additional file 1: Figure 1). Hospitals in Connecticut (47.6%), Alaska (45.5%) and North Carolina (41.8%) had the highest rates of telepsychiatry adoption. No hospitals in Delaware reported telepsychiatry.

**Table 1:** Hospital and county-level characteristics by telepsychiatry adoption in 2017

Characteristics	Number (%) of Hospitals	Number (%) of Hospitals had Telepsychiatry	Number (%) of Hospitals without Telepsychiatry	***P***
**Nationally**	3475 (100.0)	548 (15.8)	2927 (84.2)	
**Hospital Location**				
Urban	2046 (58.9)	397 (19.4)	1649 (80.6)	**< 0.001**
Rural Micropolitan	602 (17.3)	82 (13.6)	520 (86.4)	**< 0.001**
Rural Noncore	827 (23.8)	69 (8.3)	758 (91.7)	**< 0.001**
**Provision of Psychiatric Services**				
None of Inpatient and Outpatient Psychiatric Services	1526 (43.9)	104 (6.8)	1422 (93.2)	**< 0.001 < 0.001**
Inpatient Psychiatric Services Only	111 (3.2)	9 (7.9)	102 (92.1)	**0.02**
Outpatient Psychiatric Services Only	814 (23.4)	124 (15.2)	690 (84.8)	0.63
Both Inpatient and Outpatient Psychiatric Services	1024 (29.5)	311 (30.4)	713 (69.6)	**< 0.001**
**Ownership**				
Federal	55 (1.6)	44 (80.0)	11 (20.0)	**< 0.001**
Non-federal Public	731 (21.0)	92 (12.6)	639 (87.4)	**< 0.01**
Non-profit, Private	384 (11.1)	33 (8.6)	351 (91.4)	**< 0.001**
For-profit, Private	2305 (66.3)	379 (16.4)	1926 (83.6)	0.13
**System Affiliation**				**< 0.001**
Yes	2337 (67.3)	423 (18.1)	1914 (81.9)	
No	1138 (32.7)	125 (11.0)	1013 (89.0)	
**Teaching Status**				**< 0.001**
Yes	1508 (43.4)	341 (22.6)	1167 (77.4)	
No	1967 (56.6)	207 (10.5)	1760 (89.5)	
**Critical Access Hospital**				**< 0.001**
Yes	1003 (28.9)	81 (8.1)	922 (91.9)	
No	2472 (71.1)	467 (18.9)	2005 (81.1)	
**Hospital Beds Staffed**				
1–25	1034 (29.8)	76 (7.4)	958 (92.7)	**< 0.001**
26–100	800 (23.0)	112 (14.0)	688 (86.0)	0.12
101–225	747 (21.5)	128 (17.1)	619 (82.9)	0.25
> 225	894 (25.7)	232 (26.0)	662 (74.1)	**< 0.001**
**Ratio of Medicaid Inpatient Days to Total Inpatient Days**				
≤ 7.76%	887 (25.5)	105 (11.8)	782 (88.2)	**< 0.001**
7.76%-16.67	1048 (30.2)	134 (12.8)	914 (87.2)	**< 0.01**
16.67–23.61%	729 (21.0)	131 (18.0)	598 (82.0)	0.07
> 23.61%	811 (23.3)	178 (22.0)	633 (78.0)	**< 0.001**
**Profit Margins**				
Negative Margins	879 (25.3)	104 (11.8)	775 (88.2)	**< 0.001**
Positive Margins	2047 (58.9)	314 (15.3)	1733 (84.7)	0.41
Missing	549 (15.8)	130 (23.7)	419 (76.3)	**< 0.001**
**County-level Population by Age Groups, (Mean, Standard Deviation)**				
< 15	19.0% (0.026)	19.0% (0.026)	18.7% (0.026)	0.13
15–24	13.3% (0.031)	13.2% (0.031)	13.7% (0.031)	**< 0.001**
25–44	27.4% (0.036)	27.2% (0.036)	28.3% (0.036)	**< 0.001**
45–64	26.3% (0.029)	26.4% (0.030)	25.9% (0.029)	**< 0.001**
65–74	7.5% (0.019)	7.6% (0.019)	7.1% (0.019)	**< 0.001**
> 75	6.6% (0.021)	6.6% (0.021)	6.2% (0.021)	**< 0.001**
**County-level Population by Race/Ethnicity, % (Mean, Standard Deviation)**				
Non-Hispanic White	69.9% (0.219)	70.3% (0.220)	67.5% (0.213)	**< 0.001**
Non-Hispanic Black	5.3% (0.064)	5.1% (0.064)	6.1% (0.063)	**< 0.001**
American Indian and Alaska Native	1.8% (0.052)	1.8% (0.052)	16.2% (0.054)	**0.03**
Hispanic	13.3% (0.155)	13.3% (0.158)	13.0% (0.138)	**0.02**
Other	9.8% (0.094)	9.4% (0.093)	11.8% (0.976)	**< 0.001**
**County-level Population Uninsured, %**				
≤ 7.4%	1132 (32.6)	203 (17.9)	929 (82.1)	**0.02**
7.4–10.6%	926 (26.6)	153 (16.5)	773 (83.5)	0.46
10.6–14.5%	717 (20.6)	123 (17.2)	594 (82.9)	0.25
> 14.5%	700 (20.1)	69 (9.9)	631 (90.1)	**< 0.001**
**County-level Population Living in Poverty (< 200% Federal Poverty Level), %**				
≤ 26.43%	1063 (30.6)	199 (18.7)	864 (81.3)	**< 0.01**
26.43–32.58%	1064 (30.6)	164 (15.4)	900 (84.6)	0.70
32.58–39.20%	812 (23.4)	124 (15.3)	688 (84.7)	0.66
> 39.20%	536 (15.4)	61 (11.4)	475 (88.6)	**< 0.01**
**County-level Population Unemployed, %**				
≤ 3.5%	881 (25.4)	123 (14.0)	758 (86.0)	0.09
3.5–4.4%	1070 (30.8)	185 (17.3)	885 (82.7)	0.10
4.4–5.5%	976 (28.1)	158 (16.2)	818 (83.8)	0.67
> 5.5%	548 (15.8)	82 (15.0)	466 (85.0)	0.57
**Designation as a Mental Health Professional Shortage Area**				
No	218 (6.3)	48 (22.0)	170 (78.0)	**< 0.01**
Part	1676 (48.2)	334 (19.9)	1342 (80.1)	**< 0.001**
Whole	1581 (45.5)	166 (10.5)	1415 (89.5)	**< 0.001**
**County-level Total Number of Psychiatrists**				
None	1308 (37.6)	125 (9.6)	1183 (90.4)	**< 0.001**
1–4	400 (11.5)	55 (13.8)	345 (86.3)	**< 0.001**
> 4	1767 (50.8)	368 (20.8)	1399 (79.2)	**< 0.001**
**Census Region**				
Northeast	612 (17.6)	102 (16.7)	510 (83.3)	0.50
South	1146 (33.0)	167 (14.6)	979 (85.4)	0.17
Midwest	1271 (36.6)	178 (14.0)	1093 (86.0)	**0.03**
West	446 (12.8)	101 (22.7)	345 (77.3)	**< 0.001**

Notes: The *P* values are derived from Pearson’s Chi-squared tests for the categorical characteristics (percentages) and from Kruskal-Wallis rank-sum tests for the numeric characteristics for the null hypothesis that hospitals with and without telepsychiatry are the same

### Multivariate analysis of telepsychiatry adoption

As shown in Table 2, after controlling for key covariates, rural micropolitan and rural noncore hospitals no longer differed from urban hospitals in telepsychiatry adoption. Hospitals with outpatient psychiatric services only (marginal differences [95% CI]: 6.5% [3.7% to 9.4%]), as well as hospitals that offered both outpatient and inpatient psychiatric care services (16.0% [12.1% to 19.9%]) had greater likelihood of telepsychiatry adoption than hospitals without designated psychiatric services. Compared to non-federal public hospitals, federal hospitals (48.9% [32.5% to 65.3%]) were more likely to have telepsychiatry, while private non-profit hospitals (− 6.9% [− 11.7% to − 2.0%) were less likely to have telepsychiatry. System affiliated hospitals (3.9% [1.2% to 6.6%]), large hospitals (Quartile IV of hospital beds staffed vs. Quartile I: 6.2% [0.7% to 11.6%]), hospitals with greater ratio of Medicaid inpatient days to total inpatient days (4.4% [0.1% to 8.6%]), and hospitals in counties with greater proportion of population aged 25–44 years (7.2% [0.4% to 14.0%]) also reported greater likelihoods of having telepsychiatry. However, hospitals in mental health shortage counties were less likely to adopt telepsychiatry. Profit margins, county-level number of psychiatrists, racial distribution of residents, rate of uninsured residents, and US census region were not independently associated with telepsychiatry adoption in 2017.

**Table 2:** Marginal differences of hospital and county-level characteristics on telepsychiatry adoption

Characteristics	Average Marginal Differences	95% CI		***P***
**HOSPITAL CHARACTERISTICS**				
**Hospital Location**				
Urban	Ref			
Rural Micropolitan	0.1%	−4.0%	4.3%	0.95
Rural Noncore	0.7%	−4.6%	6.0%	0.79
**Provision of Psychiatric Services**				
None of Inpatient and Outpatient Psychiatric Services	Ref			
Inpatient Psychiatric Services Only	1.1%	−5.1%	7.2%	0.73
Outpatient Psychiatric Services Only	6.5%	3.7%	9.4%	**< 0.001**
Both Inpatient and Outpatient Psychiatric Services	16.0%	12.1%	19.9%	**< 0.001**
**Ownership**				
Non-federal Public	Ref			
Private For-Profit	−1.4%	−5.2%	2.4%	0.46
Private Non-Profit	−6.9%	−11.7%	−2.0%	**< 0.01**
Federal Hospitals	48.9%	32.5%	65.3%	**< 0.001**
**System Affiliation**				
No	Ref			
Yes	3.9%	1.2%	6.6%	**< 0.01**
**Hospital Beds Staffed**				
1–25	Ref			
26–100	2.4%	−1.7%	6.4%	0.25
101–225	2.1%	−2.7%	6.9%	0.39
> 225	6.2%	0.7%	11.6%	**0.03**
**Ratios of Medicaid Inpatient Days to Total Inpatient Days**				
≤ 7.76%	Ref			
7.76 -16.67%	1.6%	−2.1%	5.2%	0.40
16.67 -23.61%	3.1%	−1.4%	7.6%	0.18
> 23.61%	4.9%	0.3%	9.4%	**0.04**
**Profit Margins**				
Negative Margins	Ref			
Positive Margins	1.0%	−1.8%	3.8%	0.49
Missing	4.4%	−0.2%	9.0%	0.06
**COUNTY LEVEL CHARACTERISTICS**				
**County-level Population by Age Groups, Years** ^a^				
< 15	Ref			
15–24	2.7%	−3.9%	9.4%	0.42
25–44	7.2%	0.4%	14.0%	**0.04**
45–64	1.3%	−8.2%	10.8%	0.79
65–74	4.1%	−13.4%	21.7%	0.65
> 75	1.1%	− 12.8%	15.0%	0.88
**County-level Population by Race/Ethnicity %** ^b^				
Non-Hispanic White	Ref			
Non-Hispanic Black	−1.4%	−4.5%	1.8%	0.39
American Indian and Alaska Native	−0.7%	−4.4%	3.1%	0.73
Hispanic	−1.0%	−2.1%	0.1%	0.08
Other	−0.4%	−2.5%	1.7%	0.72
**County-level Population Uninsured, %**				
≤ 7.4%	Ref			
7.4–10.6%	1.6%	−1.4%	4.6%	0.29
10.6–14.5%	3.5%	−0.7%	7.6%	0.10
> 14.5%	2.0%	−3.9%	7.9%	0.51
**Designation as a Mental Health Professional Shortage Area**				
No	Ref			
Part	−5.2%	−11.1%	0.7%	0.08
Whole	−6.6%	−12.7%	−0.5%	**0.03**
**County-level Total Number of Psychiatrists**				
None	Ref			
1–4	−2.6%	−7.2%	1.9%	0.26
> 4	−2.7%	−7.6%	2.2%	0.28
**Census Region**				
Northeast	Ref			
South	−1.9%	−6.8%	2.9%	0.44
Midwest	−1.0%	−5.1%	3.2%	0.65
West	−1.5%	−6.6%	3.7%	0.59

Notes: Marginal differences were calculated using generalized logistic regression models that included all covariates and 95% CIs were calculated from standard errors clustered at the county level. ^a^, ^b^: the percent of population by age groups and race/ethnicity were multiplied by 10 for ease of interpretation

In the sensitivity analysis, results were robust for other hospital and county characteristics (Additional file 1: Tables 4–5). Telepsychiatry adoption rates were not different across hospitals by county-level socioeconomic characteristics.

## Discussion

Using nationwide hospital data, this study explored geographic variations in telepsychiatry adoption across US hospitals in 2017, and findings can inform efforts to improve access to psychiatric care and reduce persistent geographic disparities in mental health [17–19]. Our data indicate that less than one in six (15.8%) hospitals had telepsychiatry as of 2017. This suggests that substantial challenges remain for increasing access to psychiatric services across the US. Although the majority of rural residents live in mental health shortage areas, telepsychiatry was not routinely being used to deliver psychiatric services in these areas. Hospitals in rural noncore areas were far less likely to have adopted telepsychiatry – with only 8% of rural noncore hospitals having telepsychiatry in 2017. Telepsychiatry adoption varied significantly by both hospital- and county-level characteristics, including provision of outpatient psychiatric services, system affiliation, hospital bed size, ownership, ratio of Medicaid inpatient days to total inpatient days, and designation as mental health shortage areas.

Although telehealth has long been advocated as a tool to improve access to care and to facilitate the transition from hospital-based care to community-based care [44], we find that telepsychiatry adoption by hospitals remains very limited. More importantly, our study reveals that hospitals in counties with more psychiatrists did not have higher telepsychiatry adoption rates. Clinical resources that are clustered in certain geographic areas may have little benefit for individuals residing outside of those areas without purposeful, targeted efforts to expand access. This finding may be due to the absence of incentives, a lack of hospital buy-in, and/or limited education or training for psychiatrists to provide telepsychiatry in the hospital settings [27, 45]. In addition, many patients may face barriers to engaging in such services, particularly due to the persistent rural-urban disparities in high speed Internet access [46].

It is concerning that hospitals in rural counties have lower rates of telepsychiatry adoption, especially hospitals in noncore areas. This finding is somewhat in contrast to previous research that showed that mental health facilities in rural noncore areas have greater rates of telepsychiatry than their urban counterparts [32]; however, it is possible that disconnect exists between rural hospitals and rural mental health centers and that administrators of local hospitals have not been sufficiently motivated to expand telepsychiatry. One prior study has revealed that around 40% of individuals who died by suicide had received care within 30 days of their suicide [47]. In addition, poor continuity of care and lack of follow-up for individuals discharged from psychiatric inpatient settings are major issues [7, 48]. Providing follow-up after psychiatric hospitalization discharge has proven useful to reduce risk of non-adherence to medication and suicide [13, 14]. Telepsychiatry may facilitate timely delivery of follow-up care after discharge and make it easier to support patients’ adherence to treatment [16, 17].

Bridging these gaps calls for a wider availability of telepsychiatry to improve continuity of care. Our study reveals that hospitals with inpatient psychiatric services but without outpatient psychiatric services did not report greater adoption of telepsychiatry than their counterparts. This may be related to the per diem prospective payment system for inpatient psychiatric facilities and insufficient payment for outpatient services [49, 50]. Also, telepsychiatry adoption rates vary tenfold by facility operation, with 80% of federal hospitals but only 16% of non-federal public and 8.6% of private non-profit hospitals reporting telepsychiatry. This result is likely due to the significant progress that has been made by the Veteran Affairs system in promoting telehealth [51]. To ensure access to psychiatric care for all, federal and state policymakers should expand the types of providers eligible to receive reimbursements for both live video and remote patient monitoring for patients in need.

Our study demonstrates that hospitals with a greater ratio of Medicaid inpatient days to total inpatient days were more likely to have telepsychiatry. This suggests that federal Medicaid policies could possibly promote telepsychiatry adoption in these hospitals; surprisingly, profit margins were not independent factors associated with telepsychiatry use, even though investing in telehealth systems is perceived as a way to increase the competitive advantage of a hospital [52]. This might be related to decreasing trends in average reimbursement for telepsychiatry [53]. In 2018, over 10 states still did not have parity legislation in place for private insurance coverage of telehealth [54]; these telepsychiatry disparities are likely historically rooted, in part, in regulation and reimbursement policies. Policies to improve access to care through expanded telehealth are evolving quickly as a result of the COVID-19 pandemic; however, the public and private funding sources that will be needed for expanded telehealth remain unclear [55].

With increasing demands for psychiatric services, our findings on lower rates of telepsychiatry adoption in counties designated as mental health professional shortage areas raise concerns about access to care for residents in these already low resource areas. Lack of telepsychiatry adoption in vulnerable communities is likely compounded by the limited supply of mental health professionals to begin with. Without purposeful state and federal efforts to address the inequitable distribution of mental health resources, disparities in access to care are likely to persist. These results call for allocating telepsychiatry funding based on local mental health care need. Otherwise, residents in these counties will be less likely to have access to evidence-based treatments for mental health disorders, and the health disparities affecting the rural US are likely to persist or even worsen.

This study has some limitations. First, the AHA Annual Survey asked about hospital-wide use of telepsychiatry via a single item without querying the extent of use or scope of services offered. About 25% of hospitals did not respond to the item on telepsychiatry. Assuming that hospitals without any telehealth tend not to respond to telehealth questions, the current national rate of telepsychiatry adoption may be overestimated. Second, our cross-sectional data did not allow us to make causal inferences, and we had no data about local psychiatric care needs. Third, our study focused on the telepsychiatry adoption at hospital settings, which include EDs, as well as inpatient and outpatient psychiatric services. We did not include mental health facilities in our analysis though they often provide a broad range of services [56]. Finally, this study documented telepsychiatry availability prior to COVID-19, which might have experienced uptick due to temporary waivers by the Centers for Medicare and Medicaid Services on originating sites for telehealth and the ability of healthcare professionals to prescribe remotely during the COVID-19 pandemic [57]. Future research is warranted to study how these policy waivers impacted telepsychiatry availabilities when nationwide hospital data on telepsychiatry during 2020 are made available.

## Conclusions

This study is the first to examine the national geographic distribution of telepsychiatry adoption across US hospitals and the hospital characteristics that were associated with adoption. Significant regional and rural-urban disparities of hospital-based telepsychiatry adoption exist. Understanding the distribution of telepsychiatry adoptions and associated factors is vital to enact targeted policies to improve access to hospital-based inpatient and outpatient psychiatric care for those in need. This study found that factors related to both hospital capacity and external environments were important predictors of telepsychiatry adoption. Our results suggest that rural, isolated, small, and freestanding hospitals face disproportionate difficulties in adopting telepsychiatry. Given well-documented benefits of telepsychiatry, policies and enhanced resources are needed to ensure necessary infrastructure in small and less-resourced hospitals to ensure access to telepsychiatric care among residents, especially those in mental health professional shortage areas.

## Supplementary Information

**Additional file 1.**

## Data Availability

The datasets used and analyzed during the current study are available from the corresponding author on reasonable request.

## Abbreviations

ED: Emergency department
AHA: American Hospital Association
AIC: Akaike Information Criterion
BIC: Bayesian Information Criterion
COVID-19: Coronavirus Disease 2019
