# Supplementary material for: Telepsychiatry adoption across hospitals in the United States: a cross-sectional study
Source: BMC Psychiatry. 2021 Apr 7;21:182. doi: 10.1186/s12888-021-03180-8 (PMC8025063; doi:10.1186/s12888-021-03180-8)
Supplement: Supplementary file 1 — Additional file 1. [file 12888_2021_3180_MOESM1_ESM.docx]

# **Supplementary Information**

# **Appendix Figure 1.** Telepsychiatry Adoption in 2017 by State

**Appendix Table 1.** Comparison of Hospital and County-level Characteristics Between Hospital Reported and not Reported Telepsychiatry Data

# **Appendix Table 2.** Association between Hospital Characteristics and Telepsychiatry Adoption

# **Appendix Table 3.** Associations of Hospital Characteristics and County-level Sociodemographic and Socioeconomic Characteristics with Telepsychiatry Adoption

# **Appendix Table 4.** Associations of Hospital-level, County-level Characteristics, and Census Region with Telepsychiatry Adoption

# **Appendix Table 5.** Associations of Hospital Characteristics and County-level Sociodemographic and Socioeconomic Characteristics with Telepsychiatry Adoption – Unemployment and Poverty Rates

# **Appendix Figure 1. Telepsychiatry Adoption in 2017 by State**


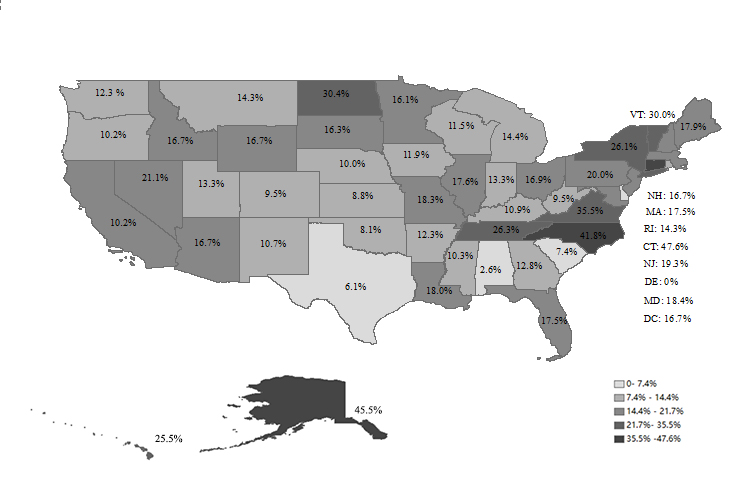


Sources: Data on telepsychiatry was derived from 2017 AHA Annual Survey dataset.

**Appendix Table 1. Comparison of Hospital and County-level Characteristics Between Hospital Reported and not Reported Telepsychiatry Data**

| **Characteristics** | **Number (%) of Hospitals** | **Number (%)**  **of Hospitals  not Reported Telepsychiatry  Data** | **Number (%)**  **of Hospitals Reported Telepsychiatry Data** | ***P*** |
| --- | --- | --- | --- | --- |
| **Nationally** | 4,602 (100.0) | 1,127 (24.5) | 3,475 (75.5) |  |
| **Hospital Location** | | | |  |
| Urban | 2,707 (58.8) | 661 (24.4) | 2,046 (75.6) | 0.89 |
| Rural Micropolitan | 769 (16.7) | 167 (21.7) | 602 (78.3) | **0.02** |
| Rural Noncore | 1,126 (24.5) | 299 (26.6) | 827 (73.5) | 0.06 |
| **Provision of Psychiatric Services** | | | |  |
| None of Inpatient and Outpatient Psychiatric Services | 2,653 (57.6) | 1,127 (42.5) | 1,526 (57.5) | **< 0.001** |
| Inpatient Psychiatric Services Only | 111 (2.4) | 0 (0) | 111 (100) | **< 0.001** |
| Outpatient Psychiatric Services Only | 814 (17.7) | 0 (0) | 814 (100) | **< 0.001** |
| Both Inpatient and Outpatient Psychiatric Services | 1,024 (22.3) | 0 (0) | 1,024 (100) | **< 0.001** |
| **Ownership** | | | |  |
| Federal | 193 (4.2) | 138 (71.5) | 55 (28.5) | **< 0.001** |
| Non-federal Public | 963 (20.9) | 232 (24.1) | 731 (75.9) | 0.75 |
| Non-profit, Private | 703 (15.3) | 319 (45.4) | 384 (54.6) | **< 0.001** |
| For-profit, Private | 2,743 (59.6) | 438 (16.0) | 2,305 (84.0) | **< 0.001** |
| **System Affiliation** | | | | 0.06 |
| Yes | 3,060 (66.5) | 723 (23.6) | 2,337 (76.4) |  |
| No | 1,542 (33.5) | 404 (26.2) | 1,138 (73.8) |  |
| **Teaching Status** | | | | **< 0.001** |
| Yes | 1,918 (41.7) | 410 (21.4) | 1,508 (78.6) |  |
| No | 2,684 (58.3) | 717 (26.7) | 1,967 (73.3) |  |
| **Critical Access Hospital** | | | | 0.97 |
| Yes | 1,329 (28.9) | 326 (24.5) | 1,003 (75.5) |  |
| No | 3,273 (71.1) | 801 (24.5) | 2,472 (75.5) |  |
| **Hospital Beds Staffed** | | | |  |
| 1-25 | 1,326 (28.8) | 292 (22.0) | 1,034 (78.0) | **0.01** |
| 26-100 | 1,149 (25.0) | 349 (30.4) | 800 (69.6) | **< 0.001** |
| 101-225 | 1,037 (22.5) | 290 (28.0) | 747 (72.0) | **0.003** |
| > 225 | 1,090 (23.7) | 196 (18.0) | 894 (82.0) | **< 0.001** |
| **Proportion of Medicaid Inpatient Days** | | | |  |
| ≤ 7.76% | 1,141 (24.8) | 330 (28.9) | 811 (71.1) | **< 0.001** |
| 7.76%%-16.67% | 1,142 (24.8) | 413 (36.2) | 729 (63.8) | **< 0.001** |
| 16.67%-23.61% | 1,160 (25.2) | 112 (9.7) | 1,048 (90.3) | **< 0.001** |
| > 23.61% | 1,159 (25.2) | 272 (23.5) | 887 (76.5) | 0.35 |
| **Profit Margins** |  | | |  |
| Negative Margins | 1160 (25.2) | 281 (24.2) | 879 (75.8) | 0.85 |
| Positive Margins | 2502 (54.4) | 455 (18.2) | 2,047 (81.8) | **< 0.001** |
| Missing | 940 (20.4) | 391 (41.6) | 549 (58.4) | **< 0.001** |
| **County-level Population by Age Groups, Years (Mean, Standard Deviation)** | | | |  |
| ≤15 | 19.0% (0.026) | 19.0% (0.028) | 19.0% (0.026) | 0.41 |
| 15-24 | 13.3% (0.030) | 13.3% (0.030) | 13.3% (0.031) | 0.57 |
| 25-44 | 27.3% (0.036) | 27.3% (0.037) | 27.4% (0.036) | 0.94 |
| 45-64 | 26.3% (0.030) | 26.2% (0.031) | 26.3% (0.029) | 0.51 |
| 65-74 | 7.5% (0.020) | 7.7% (0.022) | 7.5% (0.019) | 0.17 |
| >75 | 6.6% (0.022) | 6.5% (0.023) | 6.6% (0.021) | 0.11 |
| **County-level Population by Race/Ethnicity, % (Mean, Standard Deviation)** | | | |  |
| Non-Hispanic White | 69.0 % (0.222) | 66.2% (0.230) | 69.9% (0.219) | **< 0.001** |
| Non-Hispanic Black | 5.3% (0.064) | 5.6% (0.065) | 5.3% (0.064) | 0.23 |
| American Indian and Alaska Native | 2.1 % (0.068) | 3.1% (0.103) | 1.8% (0.052) | **< 0.001** |
| Hispanic | 13.5% (0.156) | 14.3% (0.159) | 13.3% (0.155) | 0.05 |
| Other | 10.0% (0.097) | 10.7% (.105) | 9.8% (0.944) | **< 0.01** |
| **County-level Population Uninsured, %** | | | |  |
| ≤ 7.4% | 1,414 (30.7) | 282 (19.9) | 1,132 (80.1) | **< 0.001** |
| 7.4%-10.6% | 1,272 (27.6) | 346 (27.2) | 926 (72.8) | **< 0.01** |
| 10.6%-14.5% | 1,007 (21.9) | 290 (28.8) | 717 (71.2) | **< 0.001** |
| > 14.5% | 909 (19.8) | 209 (23.0) | 700 (77.0) | 0.16 |
| **County-level Population Living in Poverty (≤ 200 % Federal Poverty Level), %** | | | |  |
| ≤ 26.43% | 1,344 (29.2) | 281 (20.9) | 1,063 (79.1) | **< 0.001** |
| 26.43%-32.58% | 1,410 (30.6) | 346 (24.5) | 1,064 (75.5) | 0.96 |
| 32.58%-39.20% | 1,071 (23.3) | 259 (24.2) | 812 (75.8) | 0.79 |
| > 39.20% | 777 (16.9) | 241 (31.0) | 536 (69.0) | **< 0.001** |
| **County-level Population Unemployed, %** | | | |  |
| ≤ 3.5% | 1,102 (23.9) | 221 (20.1) | 881 (80) | **< 0.001** |
| 3.5%-4.4% | 1,367 (29.7) | 297 (21.7) | 1,070 (78.3) | **0.005** |
| 4.4%- 5.5% | 1,317 (28.6) | 341 (25.9) | 976 (74.1) | 0.16 |
| > 5.5% | 816 (17.7) | 268 (32.8) | 548 (67.2) | **< 0.001** |
| **Designation as a Mental Health Professional Shortage Area** | | | |  |
| No | 283 (6.1) | 65 (23.0) | 218 (77.0) | 0.54 |
| Part | 2,224 (48.3) | 548 (24.6) | 1,676 (75.4) | 0.82 |
| Whole | 2,095 (45.5) | 514 (24.5) | 1,581 (75.5) | 0.95 |
| **County-level Total Number of Psychiatrists** | | | |  |
| None | 1,745 (37.9) | 437 (25.0) | 1,308 (75.0) | **< 0.001** |
| 1-4 | 496 (10.8) | 96 (19.4) | 400 (80.7) | **< 0.001** |
| > 4 | 2,361 (51.3) | 594 (25.2) | 1,767 (74.8) | 0.57 |
| **Census Region** | | | |  |
| Northeast | 561 (12.2) | 115 (20.5) | 446 (79.5) | **0.02** |
| South | 1,727 (37.5) | 456 (26.4) | 1,271 (73.6) | **0.02** |
| Midwest | 1,381 (30.0) | 235 (17.0) | 1,146 (83.0) | **< 0.001** |
| West | 933 (20.3) | 321 (34.4) | 612 (65.6) | **< 0.001** |

# **Appendix Table 2. Association between Hospital Characteristics and Telepsychiatry Adoption**

| **Characteristic** | **AOR** | **95% CI** | | ***P*** |
| --- | --- | --- | --- | --- |
| **HOSPITAL CHARACTERISTICS** | | | | |
| **Hospital Location** |  |  |  |  |
| Urban | Ref |  |  |  |
| Rural Micropolitan | 1.01 | 0.73 | 1.39 | 0.97 |
| Rural Noncore | 1.03 | 0.70 | 1.50 | .89 |
| **Provision of Psychiatric Services** | | | |  |
| None of Inpatient and Outpatient Psychiatric Services | Ref |  |  |  |
| Inpatient Psychiatric Services Only | 1.17 | 0.56 | 2.47 | 0.68 |
| Outpatient Psychiatric Services Only | 1.98 | 1.49 | 2.65 | **< 0.001** |
| Both Inpatient and Outpatient Psychiatric Services | 3.92 | 2.89 | 5.32 | **< 0.001** |
| **Ownership** | | | |  |
| Non-federal Public | Ref |  |  |  |
| Private For-Profit | 0.88 | 0.65 | 1.19 | 0.41 |
| Private Non-Profit | 0.48 | 0.30 | 0.78 | **< 0.01** |
| Federal Hospitals | 13.15 | 5.96 | 29.00 | **< 0.001** |
| **System Affiliation** | | | |  |
| No | Ref |  |  |  |
| Yes | 1.45 | 1.13 | 1.86 | **< 0.01** |
| **Hospital Beds Staffed** | | | |  |
| 1-25 | Ref |  |  |  |
| 26-100 | 1.22 | 0.83 | 1.79 | 0.32 |
| 101-225 | 1.12 | 0.71 | 1.74 | 0.63 |
| > 225 | 1.51 | 0.96 | 2.37 | 0.07 |
| **Proportion of Medicaid Inpatient Days** | | | |  |
| ≤ 7.76 % | Ref |  |  |  |
| 7.76 %-16.67 % | 1.18 | 0.83 | 1.67 | 0.36 |
| 16.67 %-23.61 % | 1.33 | 0.89 | 1.97 | 0.16 |
| > 23.61 % | 1.48 | 1.01 | 2.18 | 0.05 |
| **Profit Margins** | | | |  |
| Negative Margins | Ref |  |  |  |
| Positive Margins | 1.11 | 0.87 | 1.43 | 0.40 |
| Missing | 1.35 | 0.97 | 1.86 | 0.07 |
| **AIC-value** | 0.773 |  |  |  |
| **BIC-value** | -25535.38 | | | |

Abbreviation: AOR, adjusted odds ratio; AIC, Akaike Information Criterion; BIC, Bayesian Information Criterion.

# **Appendix Table 3. Associations of Hospital Characteristics and County-level Sociodemographic and Socioeconomic Characteristics with Telepsychiatry Adoption**

| **Characteristics** | **AOR** | **95% CI** | | ***P*** |
| --- | --- | --- | --- | --- |
| **HOSPITAL CHARACTERISTICS** | | | | |
| **Hospital Location** |  |  |  |  |
| Urban | Ref |  |  |  |
| Rural Micropolitan | 1.02 | 0.71 | 1.46 | 0.93 |
| Rural Noncore | 1.07 | 0.68 | 1.67 | 0.77 |
| **Provision of Psychiatric Services** | | | |  |
| None of Inpatient and Outpatient Psychiatric Services | Ref |  |  |  |
| Inpatient Psychiatric Services Only | 1.15 | 0.54 | 2.45 | 0.71 |
| Outpatient Psychiatric Services Only | 1.99 | 1.48 | 2.66 | **< 0.001** |
| Both Inpatient and Outpatient Psychiatric Services | 3.80 | 2.77 | 5.21 | **< 0.001** |
| **Ownership** | | | |  |
| Non-federal Public | Ref |  |  |  |
| Private For-Profit | 0.90 | 0.66 | 1.21 | 0.48 |
| Private Non-Profit | 0.51 | 0.31 | 0.84 | **< 0.01** |
| Federal Hospitals | 13.75 | 5.89 | 32.10 | **< 0.001** |
| **System Affiliation** | | | |  |
| No | Ref |  |  |  |
| Yes | 1.41 | 1.10 | 1.82 | **< 0.01** |
| **Hospital Beds Staffed** | | | |  |
| 1-25 | Ref |  |  |  |
| 26-100 | 1.25 | 0.85 | 1.84 | 0.26 |
| 101-225 | 1.21 | 0.77 | 1.92 | 0.41 |
| > 225 | 1.69 | 1.05 | 2.73 | **0.03** |
| **Proportion of Medicaid Inpatient Days** | | | |  |
| ≤ 7.76 % | Ref |  |  |  |
| 7.76 %-16.67 % | 1.16 | 0.81 | 1.66 | 0.42 |
| 16.67 %-23.61 % | 1.32 | 0.88 | 2.00 | 0.18 |
| > 23.61 % | 1.53 | 1.02 | 2.29 | **0.04** |
| **Profit Margins** | | | |  |
| Negative Margins | Ref |  |  |  |
| Positive Margins | 1.08 | 0.84 | 1.40 | 0.54 |
| Missing | 1.39 | 0.97 | 1.99 | 0.07 |
| **COUNTY LEVEL CHARACTERISTICS** | | | |  |
| **County-level Population by Age Groups, Years** * | | | | |
| <15 | Ref |  |  |  |
| 15-24 | 1.30 | 0.74 | 2.28 | 0.37 |
| 25-44 | 1.89 | 1.06 | 3.38 | **0.03** |
| 45-64 | 1.19 | 0.53 | 2.68 | 0.68 |
| 65-74 | 1.24 | 0.29 | 5.42 | 0.77 |
| >75 | 1.27 | 0.41 | 3.95 | 0.68 |
| **County-level Population by Race/Ethnicity %** † | | | |  |
| Non-Hispanic White | Ref |  |  |  |
| Non-Hispanic Black | 0.89 | 0.70 | 1.13 | 0.34 |
| American Indian and Alaska Native | 0.95 | 0.69 | 1.31 | 0.76 |
| Hispanic | 0.92 | 0.85 | 1.01 | 0.09 |
| Other | 0.96 | 0.81 | 1.14 | 0.62 |
| **County-level Population Uninsured, %** | | | |  |
| ≤ 7.4% | Ref |  |  |  |
| 7.4%-10.6% | 1.14 | 0.87 | 1.50 | 0.35 |
| 10.6%-14.5% | 1.30 | 0.93 | 1.81 | 0.12 |
| > 14.5% | 1.11 | 0.70 | 1.76 | 0.64 |
| **Designation as a Mental Health Professional Shortage Area** | | | |  |
| No | Ref |  |  |  |
| Part | 0.68 | 0.45 | 1.03 | 0.07 |
| Whole | 0.59 | 0.38 | 0.92 | **0.02** |
| **County-level Total Number of Psychiatrists** | | | |  |
| None | Ref |  |  |  |
| 1-4 | 0.81 | 0.55 | 1.19 | 0.28 |
| >4 | 0.81 | 0.54 | 1.21 | 0.30 |
| **AIC-value** | 0.777 |  |  |  |
| **BIC-value** | -25423.97 |  |  |  |

Abbreviation: AOR, adjusted odds ratio; AIC, Akaike Information Criterion; BIC, Bayesian Information Criterion. *, †: the percent of population by age groups and race/ethnicity were multiplied by 10 for ease of interpretation.

# **Appendix Table 4. Associations of Hospital-level, County-level Characteristics, and Census Region with Telepsychiatry Adoption**

| **Characteristics** | **AOR** | **95% CI** | | ***P* value** |
| --- | --- | --- | --- | --- |
| **HOSPITAL CHARACTERISTICS** | | | | |
| **Hospital Location** |  |  |  |  |
| Urban | Ref |  |  |  |
| Rural Micropolitan | 1.01 | 0.70 | 1.45 | 0.95 |
| Rural Noncore | 1.06 | 0.68 | 1.67 | 0.79 |
| **Provision of Psychiatric Services** | | | |  |
| None of Inpatient and Outpatient Psychiatric Services | Ref |  |  |  |
| Inpatient Psychiatric Services Only | 1.15 | 0.54 | 2.43 | 0.72 |
| Outpatient Psychiatric Services Only | 1.97 | 1.47 | 2.64 | **< 0.001** |
| Both Inpatient and Outpatient Psychiatric Services | 3.76 | 2.73 | 5.17 | **< 0.001** |
| **Ownership** | | | |  |
| Non-federal Public | Ref |  |  |  |
| Private For-Profit | 0.89 | 0.66 | 1.21 | 0.45 |
| Private Non-Profit | 0.52 | 0.31 | 0.85 | **.009** |
| Federal Hospitals | 13.26 | 5.67 | 31.02 | **< 0.001** |
| **System Affiliation** | | | |  |
| No | Ref |  |  |  |
| Yes | 1.42 | 1.10 | 1.83 | **< 0.01** |
| **Hospital Beds Staffed** | | | |  |
| 1-25 | Ref |  |  |  |
| 26-100 | 1.25 | 0.85 | 1.85 | 0.26 |
| 101-225 | 1.22 | 0.77 | 1.95 | 0.40 |
| > 225 | 1.70 | 1.05 | 2.76 | **0.03** |
| **Proportion of Medicaid Inpatient Days** | | | |  |
| ≤ 7.76 % | Ref |  |  |  |
| 7.76 %-16.67 % | 1.16 | 0.81 | 1.66 | 0.41 |
| 16.67 %-23.61 % | 1.32 | 0.87 | 2.01 | 0.19 |
| > 23.61 % | 1.53 | 1.01 | 2.30 | **0.04** |
| **Profit Margins** | | | |  |
| Negative Margins | Ref |  |  |  |
| Positive Margins | 1.09 | 0.85 | 1.41 | 0.50 |
| Missing | 1.44 | 0.99 | 2.09 | 0.05 |
| **COUNTY LEVEL CHARACTERISTICS** | | | |  |
| **County-level Population by Age Groups, Years*** | | | | |
| <15 | Ref |  |  |  |
| 15-24 | 1.27 | 0.71 | 2.26 | 0.42 |
| 25-44 | 1.87 | 1.04 | 3.36 | **0.04** |
| 45-64 | 1.12 | 0.49 | 2.55 | 0.79 |
| 65-74 | 1.43 | 0.31 | 6.58 | 0.65 |
| >75 | 1.10 | 0.33 | 3.67 | 0.88 |
| **County-level Population by Race/Ethnicity %** † | | | |  |
| Non-Hispanic White | Ref |  |  |  |
| Non-Hispanic Black | 0.89 | 0.68 | 1.16 | 0.39 |
| American Indian and Alaska Native | 0.94 | 0.68 | 1.31 | 0.73 |
| Hispanic | 0.92 | 0.84 | 1.01 | 0.08 |
| Other | 0.97 | 0.81 | 1.16 | 0.72 |
| **County-level Population Uninsured, %** | | | |  |
| ≤ 7.4% | Ref |  |  |  |
| 7.4%-10.6% | 1.16 | 0.88 | 1.52 | 0.29 |
| 10.6%-14.5% | 1.35 | 0.95 | 1.92 | 0.20 |
| > 14.5% | 1.19 | 0.71 | 2.00 | 0.50 |
| **Designation as a Mental Health Professional Shortage Area** | | | |  |
| No | Ref |  |  |  |
| Part | 0.67 | 0.44 | 1.02 | 0.06 |
| Whole | 0.59 | 0.38 | 0.92 | **0.02** |
| **County-level Total Number of Psychiatrists** | | | |  |
| None | Ref |  |  |  |
| 1-4 | 0.80 | 0.54 | 1.18 | 0.26 |
| >4 | 0.80 | 0.53 | 1.20 | 0.28 |
| **Census Region** |  |  |  |  |
| Northeast | Ref |  |  |  |
| South | 0.85 | 0.56 | 1.28 | 0.43 |
| Midwest | 0.92 | 0.66 | 1.30 | 0.65 |
| West | 0.88 | 0.57 | 1.37 | 0.58 |
| **AIC-value** | 0.778 |  |  |  |
| **BIC-value** | -25400.210 |  |  |  |

Abbreviation: AOR, adjusted odds ratio; AIC, Akaike Information Criterion; BIC, Bayesian Information Criterion. *, †: the percent of population by age groups and race/ethnicity were multiplied by 10 for ease of interpretation.

# **Appendix Table 5. Associations of Hospital Characteristics and County-level Sociodemographic and Socioeconomic Characteristics with Telepsychiatry Adoption – Unemployment and Poverty Rates**

| **Characteristics** | **AOR** | **95% CI** | | ***P*** | **AOR** | **95% CI** | | ***P*** |
| --- | --- | --- | --- | --- | --- | --- | --- | --- |
| **HOSPITAL CHARACTERISTICS** | | | | |  |  |  |  |
| **Hospital Location** |  |  |  |  |  |  |  |  |
| Urban | Ref |  |  |  | Ref |  |  |  |
| Rural Micropolitan | 1.04 | 0.72 | 1.50 | .82 | 1.08 | 0.74 | 1.56 | 0.69 |
| Rural Noncore | 1.11 | 0.71 | 1.74 | .65 | 1.16 | 0.73 | 1.83 | 0.54 |
| **Provision of Psychiatric Services** | | | |  |  |  |  |  |
| None of Inpatient and Outpatient Psychiatric Services | Ref |  |  |  | Ref |  |  |  |
| Inpatient Psychiatric Services Only | 1.15 | 0.54 | 2.44 | .71 | 1.17 | 0.55 | 2.47 | 0.68 |
| Outpatient Psychiatric Services Only | 1.98 | 1.47 | 2.65 | **< 0.001** | 1.95 | 1.45 | 2.61 | **< 0.001** |
| Both Inpatient and Outpatient Psychiatric Services | 3.79 | 2.76 | 5.20 | **< 0.001** | 3.75 | 2.73 | 5.16 | **< 0.001** |
| **Ownership** | | | |  |  |  |  |  |
| Non-federal Public | Ref |  |  |  | Ref |  |  |  |
| Private For-Profit | 0.88 | 0.65 | 1.19 | .41 | 0.88 | 0.65 | 1.19 | 0.34 |
| Private Non-Profit | 0.52 | 0.32 | 0.86 | **.01** | 0.53 | 0.32 | 0.86 | **0.01** |
| Federal Hospitals | 12.91 | 5.55 | 30.01 | **< 0.001** | 13.48 | 5.76 | 31.56 | **< 0.001** |
| **System Affiliation** | | | |  |  |  |  |  |
| No | Ref |  |  |  | Ref |  |  |  |
| Yes | 1.41 | 1.10 | 1.82 | **.008** | 1.43 | 1.11 | 1.84 | **< 0.01** |
| **Hospital Beds Staffed** | | | |  |  |  |  |  |
| 1-25 | Ref |  |  |  | Ref |  |  |  |
| 26-100 | 1.23 | 0.83 | 1.82 | .30 | 1.26 | 0.85 | 1.87 | 0.24 |
| 101-225 | 1.19 | 0.75 | 1.89 | .47 | 1.23 | 0.77 | 1.95 | 0.39 |
| > 225 | 1.66 | 1.02 | 2.70 | **.04** | 1.75 | 1.08 | 2.84 | 0.02 |
| **Proportion of Medicaid Inpatient Days** | | | |  |  |  |  |  |
| ≤ 7.76 % | Ref |  |  |  | Ref |  |  |  |
| 7.76 %-16.67 % | 1.15 | 0.80 | 1.64 | .45 | 1.15 | 0.81 | 1.64 | 0.44 |
| 16.67 %-23.61 % | 1.28 | 0.85 | 1.94 | .24 | 1.31 | 0.86 | 1.99 | 0.21 |
| > 23.61 % | 1.46 | 0.97 | 2.19 | .07 | 1.52 | 1.01 | 2.29 | 0.05 |
| **Profit Margins** | | | |  |  |  |  |  |
| Negative Margins | Ref |  |  |  | Ref |  |  |  |
| Positive Margins | 1.09 | 0.85 | 1.41 | .50 | 1.08 | 0.84 | 1.40 | 0.53 |
| Missing | 1.43 | 1.00 | 2.05 | .05 | 1.40 | 0.97 | 2.01 | 0.07 |
| **COUNTY LEVEL CHARACTERISTICS** | | | |  |  |  |  |  |
| **County-level Population by Age Groups, Years** * | | | | |  |  |  |  |
| <15 | Ref |  |  |  | Ref |  |  |  |
| 15-24 | 1.18 | 0.66 | 2.10 | .57 | 1.27 | 0.71 | 2.26 | 0.43 |
| 25-44 | 1.80 | 1.02 | 3.16 | **.04** | 1.73 | 0.98 | 3.07 | 0.06 |
| 45-64 | 0.95 | 0.43 | 2.10 | .90 | 0.86 | 0.39 | 1.93 | 0.72 |
| 65-74 | 1.52 | 0.33 | 7.12 | .59 | 2.29 | 0.48 | 10.98 | 0.30 |
| >75 | 1.02 | 0.30 | 3.48 | .97 | 0.89 | 0.26 | 3.02 | 0.85 |
| **County-level Population by Race/Ethnicity %** † | | | |  |  |  |  |  |
| Non-Hispanic White | Ref |  |  |  | Ref |  |  |  |
| Non-Hispanic Black | 0.92 | 0.69 | 1.23 | .57 | 1.01 | 0.76 | 1.34 | 0.94 |
| American Indian and Alaska Native | 0.95 | 0.70 | 1.29 | .74 | 0.98 | 0.72 | 1.34 | 0.91 |
| Hispanic | 0.92 | 0.84 | 1.01 | .08 | 0.94 | 0.86 | 1.02 | 0.14 |
| Other | 0.95 | 0.79 | 1.15 | .61 | 0.93 | 0.78 | 1.12 | 0.46 |
| **County-level Population Unemployed, %** | | | |  |  |  |  |  |
| ≤ 3.5% | Ref |  |  |  | Ref |  |  |  |
| 3.5%-4.4% | 1.20 | 0.89 | 1.61 | .23 |  |  |  |  |
| 4.4%- 5.5% | 1.11 | 0.82 | 1.52 | .50 |  |  |  |  |
| > 5.5% | 1.14 | 0.78 | 1.66 | .50 |  |  |  |  |
| **County-level Population Living in Poverty,**  **(< 200 % Federal Poverty Level), %** | | | |  |  |  |  |  |
| ≤ 26.43% | Ref |  |  |  | Ref |  |  |  |
| 26.43%-32.58% |  |  |  |  | 0.83 | 0.62 | 1.12 | 0.23 |
| 32.58%-39.20% |  |  |  |  | 0.93 | 0.65 | 1.31 | 0.67 |
| > 39.20% |  |  |  |  | 0.70 | 0.42 | 1.14 | 0.15 |
| **Designation as a Mental Health Professional Shortage Area** | | | |  |  |  |  |  |
| No | Ref |  |  |  | Ref |  |  |  |
| Part | 0.67 | 0.44 | 1.03 | .07 | 0.69 | 0.45 | 1.04 | 0.08 |
| Whole | 0.59 | 0.38 | 0.93 | **.02** | 0.60 | 0.39 | 0.94 | **0.02** |
| **County-level Total Number of Psychiatrists** | | | |  |  |  |  |  |
| None | Ref |  |  |  | Ref |  |  |  |
| 1-4 | 0.81 | 0.55 | 1.20 | .29 | 0.80 | 0.54 | 1.18 | 0.26 |
| >4 | 0.81 | 0.54 | 1.23 | .32 | 0.77 | 0.51 | 1.17 | 0.22 |
| **Census Region** |  |  |  |  |  |  |  |  |
| Northeast | Ref |  |  |  | Ref |  |  |  |
| South | 0.91 | 0.62 | 1.33 | .63 | 0.93 | 0.64 | 1.37 | 0.73 |
| Midwest | 0.95 | 0.67 | 1.33 | .76 | 0.94 | 0.67 | 1.33 | 0.74 |
| West | 0.97 | 0.63 | 1.49 | .88 | 0.99 | 0.64 | 1.52 | 0.95 |
| **AIC-value** | 0.779 |  |  |  | 0.778 |  |  |  |
| **BIC-value** | -25398 |  |  |  | -25400 |  |  |  |

Abbreviation: AOR, adjusted odds ratio; AIC, Akaike Information Criterion; BIC, Bayesian Information Criterion. *, †: the percent of population by age groups and race/ethnicity were multiplied by 10 for ease of interpretation.
